# *N*-thioalkylcarbazoles derivatives as new anti-proliferative agents: synthesis, characterisation and molecular mechanism evaluation

**DOI:** 10.1080/14756366.2017.1419216

**Published:** 2018-01-31

**Authors:** Maria Stefania Sinicropi, Domenico Iacopetta, Camillo Rosano, Rosario Randino, Anna Caruso, Carmela Saturnino, Noemi Muià, Jessica Ceramella, Francesco Puoci, Manuela Rodriquez, Pasquale Longo, Maria Rosaria Plutino

**Affiliations:** a Department of Pharmacy, Health and Nutritional Sciences, University of Calabria, Arcavacata di Rende, Italy;; b Biopolymers and Proteomics IRCCS Policlinico San Martino-IST, Genova, Italy;; c Department of Pharmacy, University of Salerno, Fisciano, Italy;; d Department of Science, University of Basilicata, Potenza, Italy;; e Department of Chemistry and Biology, University of Salerno, Fisciano, Italy;; f Institute for the Study of Nanostructured Materials, ISMN-CNR, Palermo, c/o Department of ChiBioFarAm, University of Messina, Messina, Italy

**Keywords:** Carbazole derivatives, apoptosis, topoisomerase II, immunofluorescence, caspases

## Abstract

Synthetic or natural carbazole derivatives constitute an interesting class of heterocycles, which showed several pharmaceutical properties and occupied a promising place as antitumour tools in preclinical studies. They target several cellular key-points, e.g. DNA and Topoisomerases I and II. The most studied representative, i.e. Ellipticine, was introduced in the treatment of metastatic breast cancer. However, because of the onset of dramatic side effects, its use was almost dismissed. Many efforts were made in order to design and synthesise new carbazole derivatives with good activity and reduced side effects. The major goal of the present study was to synthesise a series of new *N*-thioalkylcarbazole derivatives with anti-proliferative effects. Two compounds, **5a** and **5c**, possess an interesting anti-proliferative activity against breast and uterine cancer cell lines without affecting non-tumoural cell lines viability. The most active compound (**5c)** induces cancer cells death triggering the intrinsic apoptotic pathway by inhibition of Topoisomerase II.

## Graphical abstract

**Figure:**
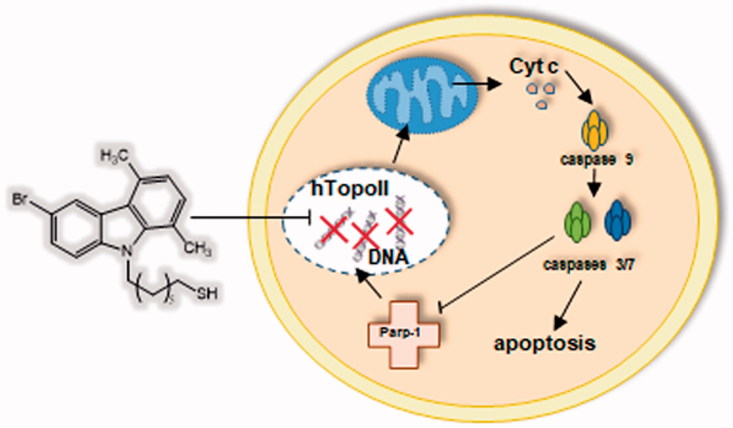

## Introduction

**Figure 1.**
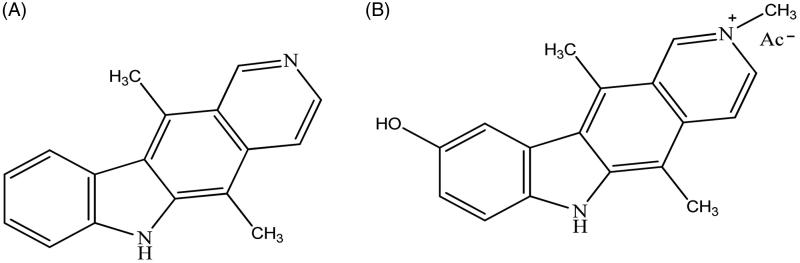
Structures of Ellipticine (A) and Celiptium (B).

Recently, one of the principal targets of the pharmaceutical research is represented by the design of new antitumoural drugs with higher selectivity on neoplastic cells, few side effects and able to overcome resistance onset^1^. In this scenario, carbazoles were widely studied since they possess not only antitumour activity but also antimicrobic, antiepileptic, antihistaminic, antioxidant, anti-inflammatory, analgesic and neuroprotective properties^2–4^. In recent studies, *N*-substituted carbazoles were reported as neuroprotective agents with strong anti-oxidant activity and some of them were also used as antifungal, photoconductive, antioxidant, antibacterial, anti-malaria, anti-tuberculosis, anti-HIV agents and in the treatment of obesity^5–7^. Regarding their antitumour activity, several key-points, namely telomerases, topoisomerase I and II, tubulin were identified^8–11^. The most studied representative of this class of compounds, i.e. Ellipticine (Figure 1) was introduced in the treatment of metastatic breast cancer^1^ ^,^ ^12–15^, then dismissed because of poor solubility in water and dramatic side effects^16^ ^,^ ^17^. An improved solubility was achieved through the formation of salts as, for instance, in the case of 9-hydroxyellipticine (Celiptium, Figure 1), which was widely used, alone or in combination with other chemotherapeutics, possesses a higher DNA affinity than Ellipticine and lacks of toxicity at therapeutic doses^18–20^.

In other previous studies, the carbazole scaffold was variously functionalised obtaining 6-aryl, 3-acyl (or 3-aroyl) and 9-alkyl-1,4-dimethyl-9*H*-carbazoles^21–26^, with improved pharmacokinetic properties. Furthermore, benzofuroquinazolinones derivatives were synthesised as carbazole bioisosters^27^ and they showed interesting antitumour properties at sub-micromolar concentrations^28^. In this paper, we report the synthesis of a new series of *N*-thioalkylcarbazoles differently substituted at the 1, 4, 6 and 9 positions of the carbazole core. In these molecules, the carbazole nitrogen bears alkyl chains with seven, eight and nine methylene groups, linked to a thiol terminal group.

Two of these molecules have demonstrated an interesting anti-proliferative activity against two breast cancer cell lines, MCF-7 and MDA-MB-231, and, to a greater extent, against cervical HeLa and endometrial ISHIKAWA cells. Moreover, the assayed compounds did not show effects on the proliferation of non-tumoural MCF-10A and 3T3-L1 cells. The antitumour activity resides in the ability to trigger the apoptotic intrinsic pathway, also known as mitochondrial pathway, and to inhibit the human Topoisomerase II (hTopo II) activity, as shown by direct enzyme assays and docking simulations. hTopoII is an essential enzymes playing a crucial role during transcription and replication and is over-expressed in tumour cells, thus it is a valid target for the development of new molecules for the treatment of a wide spectrum of tumours^29^ ^,^ ^30^. Our outcomes set the compound **5c** as a valid lead compound to be useful for cancer treatment.

## Materials and methods

### Synthesis

Commercial reagents were purchased from Sigma-Aldrich (St. Louis, MO) and used without additional purification. Silica gel 60 (300 − 400 mesh, Merck, Kenilworth, NJ) was used for flash chromatography. Preparative TLC was performed on 20 × 20 cm glass plates coated with a 2 mm layer of silica gel PF254 Merk. ^1^HNMR and ^13^CNMR spectra were recorded on a Bruker Avance 400 spectrometer (Bruker Corporation, Billerica, MA). Chemical shifts are expressed in parts per million downfield from tetramethylsilane as an internal standard. Multiplicities are represented by s (singlet), d (doublet), t (triplet), q (quartet) and m (multiplet). Coupling constants (J) are reported in Hertz (Hz). Mass spectra were obtained using an ESI mass spectrometer: LC-ADVANTAGE MAX (ESI). High-resolution mass spectra (HRMS) were recorded on a high-resolution mass spectrometer equipped by electrospray (ESI) and nanospray sources, and a quadrupole-time of flight hybrid analyser, coupled with capillary UPLC system (Q-TOF Premier/nanoAquity, Waters, Milford, MA) in positive mode, and either protonated molecular ions [M + H]^+^ were used for empirical formula confirmation, unless otherwise stated. The 9-(bromoalkyl)-9H-carbazoles **(2, 3, 4 a–c**) intermediates were prepared as described in the literature^28^ by the corresponding **1a–c** carbazoles^31–34^.

**9-(7-Bromoheptyl)-9*H*-carbazole (2a).** Colourless oil (145 mg, 71%). ^1^H NMR (400 MHz, CDCl_3_) *δ* 8.17 (d, *J* = 7.7 Hz, 2H), 7.54–7.51 (m, 2H), 7.45 (d, *J* = 8.1 Hz, 2H), 7.31–7.28 (m, 2H), 4.35–4.33 (m, 2H), 3.42–3.40 (m, 2H), 1.95–1.87 (m, 2H), 1.85–1.82 (m, 2H), 1.47–1.36 (m, 6H) ppm. HRMS (ESI-Q-TOF) *m*/*z*: Calcd. for C_19_H_22_BrN 343.0936, found 344.0939 [M + H^+^]^+^.

**9-(8-Bromooctyl)-9*H*-carbazole (3a)**. Colourless oil (129 mg, 61%). ^1^H NMR (400 MHz, CDCl_3_) *δ*: 7.51–7.37 (m, 2H), 7.36–7.22 (m, 2H), 7.17–7.12 (m, 2H), 7.11–7.06 (m, 2H), 4.10–4.06 (m, 4H), 3.37–3.33 (m, 2H), 1.86–1.82 (m, 2H), 1.68–1.64 (m, 2H), 1.40–1.29 (m, 6H) ppm. HRMS (ESI-Q-TOF) *m*/*z*: Calcd. for C_20_H_24_BrN 357.1092, found 359.1095 [M + 2]^+^.

**9-(9-Bromononyl)-9*H*-carbazole (4a)**. Colourless oil (144 mg, 65%). ^1^H NMR (400 MHz, CDCl_3_) *δ* 8.16 (d, *J* = 7.7 Hz, 2H), 7.54–7.50 (m, 2H), 7.45 (d, *J* = 8.1 Hz, 2H), 7.30–7.27 (m, 2H), 4.35–4.32 (m, 2H), 3.44–3.42 (m, 2H), 1.94–1.87 (m, 2H), 1.85–1.83 (m, 2H), 1.44–1.30 (m, 10H) ppm. HRMS (ESI-Q-TOF) *m*/*z*: Calcd. for C_21_H_26_BrN 371.1249, found 373.1245 [M + 2]^+^.

**9-(7-Bromoheptyl)-1,4,6-trimethyl-9H-carbazole (2b).** Colourless oil (75 mg, 81%). ^1^H NMR 400 MHz, CDCl_3_) *δ* 8.00 (s, 1H), 7.35–7.28 (m, 2H), 7.09 (d, *J* = 7.3 Hz, 1H), 6.90 (d, *J* = 7.3 Hz, 1H), 4.53–4.50 (m, 2H), 3.45–3.40 (m, 2H), 2.88 (s, 3H), 2.81 (s, 3H), 2.59 (s, 3H), 1.92–1.81 (m, 4H), 1.51–1.35 (m, 6H) ppm. HRMS (ESI-Q-TOF) *m*/*z*: Calcd. for C_22_H_28_BrN 385.1405, found 387.1402 [M + 2]^+^.

**9-(8-Bromooctyl)-1,4,6-trimethyl-9*H*-carbazole (3b)**. Colourless oil (70 mg, 73%). ^1^H NMR (400 MHz, CDCl_3_) *δ* 7.96 (s, 1H), 7.28–7.24 (m, 2H), 7.03 (d, *J* = 7.3 Hz, 1H), 6.85 (d, *J* = 7.3 Hz, 1H), 4.44–4.41 (m, 2H), 3.36–3.34 (m, 2H), 2.82 (s, 3H), 2.74 (s, 3H), 2.53 (s, 3H), 1.81–1.72 (m, 4H), 1.40–1.25 (m, 8H) ppm. HRMS (ESI-Q-TOF) *m*/*z*: Calcd. for C_23_H_30_BrN 399.1562, found 401.1565 [M + 2]^+^.

**9-(9-Bromononyl)-1,4,6-trimethyl-*9H*-carbazole (4b)**. Colourless oil (64 mg, 64%). ^1^H NMR (400 MHz, CDCl_3_) *δ* 8.01 (s, 1H), 7.34–7.28 (m, 2H), 7.09 (d, *J* = 7.3 Hz, 1H), 6.90 (d, *J* = 7.2 Hz, 1H), 4.52–4.49 (m, 2H), 3.44–3.41 (m, 2H), 2.87 (s, 3H), 2.80 (s, 3H), 2.58 (s, 3H), 1.89–1.78 (m, 4H), 1.58–1.28 (m, 10H) ppm. HRMS (ESI-Q-TOF) *m*/*z*: Calcd. for C_24_H_32_BrN 413.1718; found 415.1721 [M + 2]^+^.

**6-Bromo-9-(7-bromoheptyl)-1,4-dimethyl-9*H-*carbazole (2c)**. Colourless oil (102 mg, 41%). ^1^H NMR (400 MHz, CDCl_3_) *δ* 8.13 (s, 1H), 7.40–7.37 (m, 1H), 7.13 (d, *J* = 8.9 Hz, 1H), 6.96 (d, *J* = 7.3 Hz, 1H), 6.77 (d, *J* = 7.3 Hz, 1H), 4.35–4.31 (m, 2H), 3.25–3.22 (m, 2H), 2.67 (s, 3H), 2.63 (s, 3H), 1.70–1.61 (m, 4H), 1.30–1.18 (m, 6H) ppm. HRMS (ESI-Q-TOF) *m*/*z*: Calcd. for C_21_H_25_Br_2_N 449.0354; found 451.0355 [M + 2]^+^.

**6-Bromo-9-(8-bromooctyl)-1,4-dimethyl-9*H*-carbazole (3c).** Colourless oil (95 mg, 37%). ^1^H NMR (400 MHz, CDCl_3_) *δ* 8.13 (s, 1H), 7.39–7.36 (m, 1H), 7.13–7.11 (m, 1H), 6.96 (d, *J* = 7.3 Hz, 1H), 6.76 (d, *J* = 7.3 Hz, 1H), 4.32–4.28 (m, 2H), 3.29–3.23 (m, 4H), 2.66 (s, 3H), 2.62 (s, 3H), 1.75–1.63 (m, 4H), 1.29–1.17 (m, 6H). HRMS (ESI-Q-TOF) *m*/*z*: Calcd. for C_22_H_27_Br_2_N 463.0510; found 465.0513 [M + 2]^+^.

**6-Bromo-9-(9-bromononyl)-1,4-dimethyl-9*H*-carbazole (4c)**. Colourless oil (105 mg, 61%). ^1^H NMR (400 MHz, CDCl_3_) *δ* 8.13 (s, 1H), 7.38 (d, *J* = 8.6 Hz, 1H), 7.13 (d, *J* = 8.8 Hz, 1H), 6.96 (d, *J* = 7.3 Hz, 1H), 6.77 (d, *J* = 7.2 Hz, 1H), 4.33–4.30 (m, 2H), 3.25 (t, *J* = 6.8 Hz, 2H), 2.67 (s, 3H), 2.63 (s, 3H), 1.73–1.60 (m, 4H), 1.26–1.12 (m, 10H). HRMS (ESI-Q-TOF) *m*/*z*: Calcd. for C_23_H_29_Br_2_N 477,0667; found 479.1599 [M + 2]^+^.

**General procedure for the synthesis of *N*-thioalkylcarbazole derivatives (5, 6, 7a–c)**. To a mixture of the 9-(bromoalkyl)-9*H*-carbazoles **2, 3, 4a–c** (0.21 mmol) in *i*-PrOH (13 ml) thiourea (0.84 mmol) was added and the solution was refluxed for 12 h under nitrogen atmosphere^35^. At the residue obtained, after removal of the solvent, was added 6 N NaOH (27 ml), the resulting mixture was refluxed for 5 h, then it was neutralised by dropwise addition of 3 N HCl. Finally, by extraction with CH_2_Cl_2_ (3 × 100 ml), we obtained the crude compounds, purified by preparative thin layer chromatography (PTLC) (*n*-hexane/ethyl acetate 97:3) to give pure compounds.

**7-(9*H*-Carbazol-9-yl)heptane-1-thiol (5a).** Yellow oil (17 mg, 25%). ^1^H NMR (400 MHz, CDCl_3_) *δ*: 8.14 (d, *J* = 7.6 Hz, 2H, Ar), 7.51–7.48 (m, 2H, Ar), 7.43 (d, *J* = 8.1 Hz, 2H, Ar), 7.27 (m, 2H, Ar), 4.33–4.31 (m, 2H, NCH_2_), 2.66–2.63 (m, 2H, CH_2_S), 1.92–1.86 (m, 2H, NCH_2_C*H_2_*), 1.66–1.64 (m, 2H, HSCH_2_C*H_2_*), 1.41–1.37 (m, 7H, 3CH_2,_ SH) ppm. ^13^C NMR (100 MHz, CDCl_3_) *δ*: 140.73, 126.45, 124.15, 121.68, 120.48, 109.63, 47.43, 33.25, 28.96, 28.61, 27.95, 27.55, 25.38 ppm. MS(ESI): C_19_H_23_NS (298.46) [M + H^+^]^+^. HRMS (ESI-Q-TOF) *m*/*z*: Calcd. for C_19_H_23_NS 297.1551, found 298.1549 [M + H^+^]^+^.

**8-(9*H*-Carbazol-9-yl)octane-1-thiol (6a).** Yellow oil (14 mg, 23%).^1^H NMR (300 MHz, CDCl_3_) *δ*: 8.10 (d, *J* = 7.8 Hz, 2H, Ar), 7.49–7.32 (m, 4H, Ar), 7.24–7.19 (m, 2H, Ar), 4.33–4.24 (m, 2H, NCH_2_), 2.64–2.59 (m, 2H, CH_2_S), 1.87–1.82 (m, 2H NCH_2_CH_2_), 1.63–1.55 (m, 2H, HSCH_2_CH_2_), 1.47–1.25 (m, 9H, 4CH_2,_ SH) ppm. ^13^C NMR (100 MHz, CDCl_3_) *δ*: 140.73, 126.45, 124.15, 121.68, 120.48, 109.63, 47.43, 33.25, 28.96, 28.61, 27.95, 27.55, 25.38 ppm. MS (ESI): C_20_H_25_NS (312.48) [M + H^+^]^+^. HRMS (ESI-Q-TOF) *m*/*z*: Calcd. for C_20_H_25_NS 311.1708, found 312.4844 [M + H^+^]^+^.

**9-(9*H*-Carbazol-9-yl)nonane-1-thiol (7a).** Yellow oil (13 mg, 40%). ^1^H NMR (300 MHz, CDCl_3_) *δ*: 7.95 (d, *J* = 7.7 Hz, 2H, Ar), 7.33–7.24 (m, 4H, Ar), 7.11–7.06 (m, 2H Ar), 4.16–4.12 (t, 2H, NCH_2_), 2.51–2.47 (m, 2H, CH_2_S), 1.73–1.67 (m, 2H, CH_2_), 1.53–1.44 (m, 2H, CH_2_), 1.20–0.98 (m, 11H, 5CH_2_, SH) ppm. ^13^C NMR (100 MHz, CDCl_3_) *δ* 140.73, 126.45, 124.15, 121.68, 120.48, 109.63, 47.43, 33.25, 28.96, 28.61, 27.95, 27.55, 25.38 ppm. MS (ESI): C_21_H_27_NS (326.51) [M + H^+^]^+^. HRMS (ESI-Q-TOF) *m*/*z*: Calcd. for C_21_H_27_NS 325.1864, found 326.1844 [M + H^+^]^+^.

**7-(1,4,6-Trimethyl-9*H*-carbazol-9-yl)-heptane-1-thiol (5b).** Yellow oil (23 mg, 52%). ^1^H NMR (400 MHz, CDCl_3_) *δ*: 8.05 (s, 1H, Ar), 7.36–7.28 (m, 2H, Ar), 7.12 (d, *J* = 7.2 Hz, 1H, Ar), 6.94 (d, *J* = 7.2 Hz, 1H, Ar), 4.57–4.45 (m, 2H, NCH_2_), 2.83 (s, 3H, CH_3_), 2.77 (s, 3H, CH_3_), 2.74–2.71 (m, 2H, CH_2_S), 2.60 (s, 3H, CH_3_), 1.83–1.77 (m, 2H, CH_2_), 1.76–1.74 (m, 2H, CH_2_), 150–1.27 (m, 7H, 3CH_2,_ SH) ppm. ^13^C NMR (100 MHz, CDCl_3_) *δ*: 139.01, 136.81, 131.45, 130.73, 127.92, 127.11, 125.67, 123.58, 123.13, 122.63, 120.23, 111.57, 48.35, 33.25, 28.96, 28.61, 27.95, 27.55, 25.38, 21.21, 20.58, 18.02 ppm. MS (ESI): C_22_H_29_NS (340.54) [M + H^+^]^+^. HRMS (ESI-Q-TOF) *m*/*z*: [M + H]^+^ Calcd. for C_22_H_29_NS 339.2021, found 340.2017 [M + H^+^]^+^.

**8-(1,4,6-Trimethyl-9*H*-carbazol-9-yl)-octane-1-thiol (6b).** Yellow oil (14 mg, 31%). ^1^H NMR (400 MHz, CDCl_3_) *δ*: 8.07 (s, 1H, Ar), 7.36–7.34 (m, 2H, Ar), 7.14 (d, *J* = 7.2 Hz, 1H, Ar), 6.95 (d, *J* = 7.2 Hz, 1H, Ar), 4.54–4.50 (m, 2H, NCH_2_), 2.93 (s, 3H, CH_3_), 2.84 (s, 3H, CH_3_), 2.75–2.72 (m, 2H CH_2_S), 2.64 (s, 3H, CH_3_), 1.85–1.82 (m, 2H, CH_2_), 1.75–1.71 (m, 2H, CH_2_), 1.65–1.33 (m, 9H, 4 CH_2_, SH) ppm. ^13^C NMR (100 MHz, CDCl_3_) *δ*: 139.01, 136.81, 131.45, 130.73, 127.92, 127.11, 125.67, 123.58, 123.13, 122.63, 120.23, 111.57, 48.35, 33.25, 28.96, 28.61, 27.95, 27.55, 25.38, 21.21, 20.58, 18.02 ppm. MS (ESI): C_23_H_31_NS (354.56) [M + H^+^]^+^. HRMS (ESI-Q-TOF) *m*/*z*: [M + H]^+^ Calcd. for C_23_H_31_NS 353.2177, found 354.2178 [M + H^+^]^+^.

**9-(1,4,6-Trimethyl-9*H*-carbazol-9-yl)-nonane-1-thiol (7b).** Yellow oil (18 mg, 51%). ^1^H NMR (400 MHz, CDCl_3_) *δ*: 8.03 (s, 1H, Ar), 7.36–7.32 (m, 2H, Ar), 7.11 (d, *J* = 7.2 Hz, 1H, Ar), 6.92 (d, *J* = 7.2 Hz, 1H, Ar), 4.53–4.49 (m, 2H, NCH_2_), 2.90 (s, 3H, CH_3_), 2.82 (s, 3H, CH_3_), 2.73–2.70 (m, 2H, CH_2_S), 2.60 (s, 3H, CH_3_), 1.83–1.77 (m, 2H, CH_2_), 1.72–1.62 (m, 2H, CH_2_), 1.43–1.31 (m, 11H, 5CH_2_, SH) ppm. ^13^C NMR (100 MHz, CDCl_3_) *δ*: 139.01, 136.81, 131.45, 130.73, 127.92, 127.11, 125.67, 123.58, 123.13, 122.63, 120.23, 111.57, 48.35, 33.25, 28.96, 28.61, 27.95, 27.55, 25.38, 21.21, 20.58, 18.02 ppm. MS (ESI): C_24_H_33_NS (368.59) [M + H^+^]^+^. HRMS (ESI-Q-TOF) *m*/*z*: Calcd. for C_24_H_33_NS 367.2334, found 368.2336 [M + H^+^]^+^.

**7-(6-Bromo-1, 4-dimethyl-9*H*-carbazol-9-yl)-heptane-1-thiol (5c).** Yellow oil (22 mg, 48%). ^1^H NMR (400 MHz, CDCl_3_) *δ* 8.28 (s, 1H, Ar), 7.54–7.51 (m, 1H, Ar), 7.29–7.26 (m, 1H, Ar), 7.12–7.10 (m, 1H, Ar), 6.91 (d, *J* = 7.3 Hz, 1H, Ar), 4.49–4.46 (m, 2H, NCH_2_), 2.81 (s, 3H, CH_3_), 2.77 (s, 3H, CH_3_), 2.53–2.47 (m, 2H, CH_2_S), 1.81–1.77 (m, 2H, CH_2_), 1.60–1.55 (m, 2H, CH_2_), 1.41–1.25 (m, 7H, 3CH_2_, SH) ppm. ^13^C NMR (100 MHz, CDCl_3_) *δ*: 142.37, 139.01, 130.73, 128.14, 127.83, 127.11, 124.71, 123.13, 122.63, 120.23, 118.45, 112.31, 48.35, 33.25, 28.96, 28.61, 27.95, 27.55, 25.38, 20.58, 18.02 ppm. MS (ESI): C_21_H_26_BrNS (405.41) [M + 2]^+^. HRMS (ESI-Q-TOF) *m*/*z*: Calcd. for C_21_H_26_BrNS 403.0969, found 405.4066 [M + 2]^+^.

**8-(6-Bromo-1, 4-dimethyl-9*H*-carbazol-9-yl)-octane-1-thiol (6c).** Yellow oil (14 mg, 31%). ^1^H NMR (400 MHz, CDCl_3_) *δ* 8.26 (s, 1H, Ar), 7.53–7.51 (m, 1H, Ar), 7.28–7.26 (m, 1H, Ar), 7.11–7.09 (m, 1H, Ar), 6.91–6.89 (m, 1H, Ar), 4.48–4.44 (m, 2H, NCH_2_), 2.81 (s, 3H, CH_3_), 2.77 (s, 3H, CH_3_), 2.68–2.63 (m, 2H, CH_2_S), 1.80–1.76 (m, 2H, CH_2_), 1.66–1.62 (m, 2H, CH_2_), 1.36–1.30 (m, 9H, 4CH_2_, SH) ppm. ^13^C NMR (100 MHz, CDCl_3_) *δ*: 142.37, 139.01, 130.73, 128.14, 127.83, 127.11, 124.71, 123.13, 122.63, 120.23, 118.45, 112.31, 48.35, 33.25, 28.96, 28.61, 27.95, 27.55, 25.38, 20.58, 18.02 ppm. MS (ESI): C_22_H_28_BrNS (419.43) [M + 2]^+^. HRMS (ESI-Q-TOF) *m*/*z*: Calcd. for C_22_H_28_BrNS 417.1126, found 418.1129 [M + H^+^]^+^.

**9-(6-Bromo-1,4-dimethyl-9*H*-carbazol-9-yl)-nonane-1-thiol (7c).** Yellow oil (12 mg, 29%). ^1^H NMR (400 MHz, CDCl_3_) *δ* 8.26 (s, 1H, Ar), 7.53–7.51 (m, 1H, Ar), 7.28–7.26 (m, 1H, Ar), 7.11–7.09 (m, 1H, Ar), 6.91–6.89 (m, 1H, Ar), 4.46–4.44 (m, 2H, NCH_2_), 2.81 (s, 3H, CH_3_), 2.77 (s, 3H, CH_3_), 2.68–2.64 (m, 2H, CH_2_S), 1.81–1.74 (m, 2H, CH_2_), 1.69–1.65 (m, 2H, CH_2_), 1.37–1.26 (m, 11H, 5CH_2_, SH) ppm. ^13^C NMR (100 MHz, CDCl_3_) *δ* 142.37, 139.01, 130.73, 128.14, 127.83, 127.11, 124.71, 123.13, 122.63, 120.23, 118.45, 112.31, 48.35, 33.25, 28.96, 28.61, 27.95, 27.55, 25.38, 20.58, 18.02 ppm. MS (ESI): C_23_H_30_BrNS (433.46) [M + 2]^+^. HRMS (ESI-Q-TOF) *m*/*z*: Calcd. for C_23_H_30_BrNS 431.1282, found 432.1129 [M + H^+^]^+^.

### Biological assay

#### Cell cultures

The six cell lines used in this work were purchased from American Type Culture Collection (ATCC, Manassas, VA). MCF-7 human breast cancer cells, oestrogen receptor (ER) positives, were maintained in Dulbecco’s Modified Eagle’s Medium/Nutrient Mixture F-12 Ham (DMEM/F12), supplemented with 5% foetal bovine serum (FBS) and 100 U/ml penicillin/streptomycin, as previously described^36^. MDA-MB-231, human breast cancer cells, oestrogen receptor (ER) negative, were maintained in Dulbecco’s Modified Eagle’s Medium/Nutrient Mixture F-12 Ham (DMEM/F12), supplemented with 5% Newborn Calf Serum (NCS) and 100 U/ml penicillin/streptomycin. HeLa human epithelial cervix carcinoma cells, oestrogen receptor (ER) negative, and ISHIKAWA human endometrial adenocarcinoma cell, oestrogen receptor positive, were maintained in minimum essential medium (MEM), supplemented with 10% FBS, 100 U/ml penicillin/streptomycin and 1% non-essential amino acid. 3T3-L1 cells line of murine fibroblasts of embryonic type, were maintained in DMEM, supplemented with 10% NCS and 100 U/ml penicillin/streptomycin. MCF-10A human mammary epithelial cells were cultured in DMEM/F12 medium, supplemented with 5% horse serum (HS) (Eurobio, Les Ullis, Cedex, France), 100 U/ml penicillin/streptomycin, 0.5 mg/ml hydrocortisone, 20 ng/ml hEGF (human epidermal growth factor), 10 μg/ml insulin and 0.1 mg/ml cholera enterotoxin (Sigma-Aldrich, Milano, Italy). Cells were maintained at 37 °C in a humidified atmosphere of 95% air and 5% CO_2_ and periodically screened for contamination^37^.

#### Cell viability

MDA-MB-231, MCF-7, HeLa and ISHIKAWA cells were grown in complete medium and, before being treated, they were serum deprived for 24 h, to allow cell cycle synchronisation. Then cells were grown in phenol red*-*free medium supplemented with 1% DCC (destran-coated charcoal treated) FBS, then treated with increasing concentrations (0.1, 1, 10, 20, 40, 100 µM) of each compound for 72 h. Compounds were dissolved in dimethylsulphoxide (DMSO) (Sigma, St. Louis, MO) and diluted in each cell medium. Untreated cells, used as a control, were added with DMSO alone (final concentration 0.1%). Cell viability was assessed using the 3-[4,5-dimethylthiazol-2-yl]-2,5-diphenyltetrazolium bromide reagent (MTT), according to the protocol from manufacturer (Sigma-Aldrich, Milan, Italy), as previously described^37^ ^,^ ^38^. For each sample, the mean absorbance, measured at 570 nm, was expressed as a percentage of the control and plotted versus drug concentration to determine for each cell line the IC_50_ values (namely, drugs concentrations able to reduce cell viability of 50% with respect to the control), using GraphPad Prism 5 Software (GraphPad Inc., San Diego, CA)^39^. Each experiment was carried out in quadruplicate. Standard deviations (SD) were shown.

#### Cell proteins extraction and immunoblot analysis

Cells were serum deprived for 24 h, treated for 24, 48 and 72 h with the compound to be tested. At the end, cells were rinsed twice with ice cold PBS and immediately lysed in lysis buffer (50 mM Tris-HCl, pH 7, 5–8; 150 mM NaCl, 1% Igepal CA-630; 0.1% SDS; 0.5% sodium deoxycholate) containing 1% sodium orthovanadate and 1% phenylmethylsulphonyl fluoride (PMSF). Cell lysates were cleared by centrifugation at 14,000 *g* for 10 min at 4 °C. Protein concentrations were determined using the Bradford protein assay^40^ (Bio-Rad Laboratories, Milan, Italy) according to the directions from the manufacturer. Equal amounts of cell extracts (about 20 µg) were resolved under denaturing conditions by electrophoresis in 10% polyacrylamide gels containing SDS (SDS-PAGE) and transferred to nitrocellulose membranes by electroblotting (GE Healthcare, Piscataway, NJ). Membranes were first stained with Ponceau S, washed with 1× TBST (Tween-20 0,1%, Tris/HCl 10 mM e NaCl 100 mM), incubated with TBST containing 5% milk for 1 h (blocking solution) and, then, incubated overnight at 4 °C with the primary antibody raised against Parp-1(7150) or GADPH (25778), purchased from Santa Cruz-Biotechnology Inc. (Santa Cruz, CA) and used at 1:500 and 1:2000 dilutions, respectively. After incubation with the appropriate secondary antibody (VWR International PBI, MI, Italy) for 1 h at room temperature, the proteins of interest were detected on the membranes by using enhanced chemiluminescence (Amersham ECL Prime Western Blotting Detection Reagent, GE Healthcare, Piscataway, NJ).

#### TUNEL assay

Apoptosis was detected by the TUNEL assay, according to the guidelines of the manufacturer (CF™488A TUNEL Assay Apoptosis Detection Kit, Biotium, Hayward, CA)^8^. Briefly, cells were grown on glass coverslips and, after treatment, they were washed trice with PBS, then methanol-fixed at −20 °C for 15 min. Fixed cells were washed trice with 0.01% (V/V) Triton X-100 in PBS and incubated with 100 µL of TUNEL equilibration buffer for 5 min. After its removal, 50 µL of TUNEL reaction mixture containing 1 µL of terminal deoxynucleotidyl transferase (TdT) were added to each sample and incubated in a dark and humidified chamber for 2 h at 37 °C. Samples were washed trice with ice-cold phosphate-buffered saline (PBS) containing 0.1% Triton X-100 and 5 mg/mL bovine serum albumin (BSA). 2-(4-Amidinophenyl)-6-indolecarbamidine dihydrochloride (DAPI) (0.2 µg/mL) counterstain was performed (10 min, 37 °C, dark and humidified conditions). After three additional washes with cold PBS, one drop of mounting solution was added, then they were observed and imaged under a fluorescence microscope (Leica DM 6000, Leica, Frankfurt am Main, Germany) (20× magnification) with excitation/emission wavelength maxima of 490 nm/515 nm (CF^TM^488A) or 350 nm/460 nm (DAPI). Representative fields were shown. The experiments were repeated three times^41^.

#### Immunofluorescence

Cells were grown on glass coverslips in full media, then serum-deprived for 24 h and exposed to compound to test, at the indicated time. Then, they were PBS-washed, fixed with cold methanol (15 min/−20 °C) and washed three times (10 min/room temperature) with cold PBS containing 0.01% TritonX-100. After incubation (30 min/room temperature) with blocking solution (PBS, 2% BSA), they were incubated with primary antibody diluted in blocking solution (4 °C/overnight). The mouse anti-cytochrome c (556433) was purchased from BD Biosciences (Franklin Lakes, NJ) and used at 1:100 dilution. Coverslips were then washed three times with PBS, then fixed cells were incubated with the secondary antibody Alexa Fluor® 568 conjugate goat-anti-mouse (1:500, Thermo Fisher Scientific, Waltham, MA). Nuclei were stained using DAPI (Sigma-Aldrich, Milan, Italy) for 10 min at a concentration of 0.2 µg/mL then washed three times with PBS. Fluorescence was detected using a fluorescence microscope (Leica DM 6000 Leica, Frankfurt am Main, Germany). LASX software was used to acquire and process all images^42^.

#### Caspase assay

Caspases-3/7, -8 and -9 activities were measured with the Caspase-Glo Assay, according to the guidelines of manufacturer (Caspase-Glo^®^ 3/7, 8 and 9 Assay Systems, Promega Corporation, Madison, WI) as described in Iacopetta et al.^43^.

### Statistical analysis

Data were analysed for statistical significance (*p* < .001) using one-way ANOVA followed by Dunnett’s test performed by GraphPad Prism 5 (GraphPad Software, La Jolla, CA). Standard deviations (SD) are shown.

*Human topoisomerase I relaxation assay*: hTopoI relaxation assays were performed as reported in Iacopetta et al.^8^.

*Human topoisomerase II decatenation assay*: hTopoII decatenation assays were performed as reported in Iacopetta et al^.8^.

*Computer modelling and docking simulations*: The crystal structure of hTopoII in complex with a short DNA fragment and the ATP-analogue AMPPNP [PDB Code 4GFH] has been used as a template to build the complete three-dimensional models of hTopoII, as previously described^8^. The atomic coordinates of the three-dimensional model obtained were then used as a target for all the following molecular docking simulations. The ligand structures have been built and energy minimised using the program MarvinSketch [ChemAxon ltd, Budapest, Hungry]. In order to investigate the possible binding modes of **5c** to the protein, calculations were carried out with “Achilles” Blind Docking Server, available at: http://bio-hpc.eu/software/blind-docking-server/. Using a “blind docking” approach, the docking of the small molecule to the targets is done without *a priori* knowledge of the location of the binding site by the system^44^. Figures were drawn using the program Chimera^45^.

## Results and discussion

### Chemistry

**Scheme 1.**
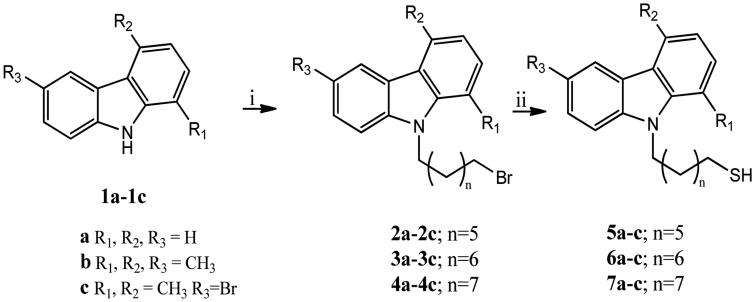
Synthesis of *N*-9*H*-alkyltiocarbazoles **5–7a–c**. Reagents and conditions: (i) 1, *N*-dibromoalkane, NaH 60%, DMF, rt; (ii) Thiourea, *i*-PrOH, reflux, 12 h; NaOH 6 N, reflux, 5 h; HCl 3 N, rt, 10 min.

The *N*-thioalkylcarbazole derivatives (**5, 6, 7a-c**) were synthesised by the reaction of appropriate 9-(bromoalkyl)-9*H*-carbazoles (**2, 3, 4a-c**) with thiourea in *i*-PrOH (at reflux for 12 h), under a nitrogen atmosphere (Scheme 1). The obtained residue, after removal of the solvent, was first treated with 6 N NaOH (reflux for 5 h), and then with 3 N HCl (at room temperature, hereafter rt). The obtained crude was purified by PTLC (*n*-hexane/ethyl acetate 97:3) to give the pure compounds. The intermediates 9-(bromoalkyl)-9*H*-carbazoles (**2, 3, 4a–c**) were prepared following general synthetic methods^28^. In particular, carbazole derivatives (**1a–c**) and dry DMF were stirred at rt until became clear. Then, NaH 60% oil dispersion and, successively, the appropriate dibromoalkane were added at 0 °C, and the mixture was stirred for 5 h at rt. Finally, water was added and the resulting mixture was extracted with EtOAc. The obtained residue was purified by silica gel column chromatography (Et_2_O/hexane, 2/3 as eluent) to give the pure compounds.

### Antiproliferative activity

All synthesised carbazole derivatives (**5a–c, 6a–c, 7a–c**) were examined to test their antiproliferative activity against two human breast cancer cell lines, namely the MCF-7 cells expressing the oestrogen receptor α (ERα), and the MDA-MB-231 triple negative cells (ER-, PR- and HER-2/Neu not amplified), and two cell lines of uterine cancer, which are the Hela cells, from uterine cervix epithelium (ER−), and endometrial ISHIKAWA cells (ER+). Cells were subjected to 72 h of continuous exposure to the compounds to test and, at the end, their viability was measured by MTT assay. Ellipticine, which represents the main natural molecule containing the carbazole moiety, was used as reference molecule for these studies. The results, summarised in Table 1, clearly indicate that only two compounds, **5a** and **5c,** possess an antitumour activity. In both breast cancer cells, a moderate antitumour activity was noticed; indeed the IC_50_ values for the compounds **5a** and **5c** in MCF-7 cells were found to be, respectively, 50.4 ± 0.96 µM and 54.9 ± 1.00 µM. In MDA-MB-231 cells, the IC_50_ values resulted lower than those determined for the MCF-7 cells and were 39.9 ± 0.52 µM and 27.2 ± 0.75 µM, respectively. However, a higher antitumour activity was found against HeLa and ISHIKAWA cells, where compound **5a** showed IC_50_ values of 12.8 ± 0.62 µM and 19.2 ± 1.13 µM, respectively. Compound **5c** showed IC_50_ values of 11.3 ± 0.63 µM (HeLa) and 15.7 ± 0.95 µM (ISHIKAWA). These data set the compound **5c** the most active and selective for uterine tumour cells. Moreover, both compounds did not affect the proliferation of MCF-10A cells, human non-malignant breast epithelial cells, and 3T3-L1 murine fibroblast-like cells, at least at doses lower than 100 µM. Finally, Ellipticine demonstrates a higher antitumour activity in all cell lines used in these assays with respect to compounds **5a** and **5c**, but together with dramatic cytotoxic effects on normal cells (Table 1). Thus, a relevant antitumour activity was obtained for compound **5c,** which hold two methyl substituents at the position 1 and 4, a bromine at position 6 and a 7 terms chain on the carbazole ring nitrogen. The replacement of bromine at position 6 with a methyl group (**5b**)^12^ ^,^ ^22^ ^,^ ^24^ ^,^ ^25^ ^,^ ^28^ and the elongation of the chain produced a loss of the antitumour activity (**6c** and **7c**). With respect to compound **5c,** a lesser antitumour activity was noticed for compound **5a**. The latter does not possess substituents on the carbazolic scaffold and holds a 7 terms chain on the nitrogen at position 9.

**Table 1.** Antiproliferative activity: IC_50_ values of carbazole derivatives (**5a–c, 6a–c, 7a–c**) and Ellipticine, expressed in micromolar (µM).

	MCF-7	MDA-MB-231	ISHIKAWA	HeLa	MCF-10a	3T3-L1
**Ellipticine**	1.25 ± 0.30	1.85 ± 0.15	1.70 ± 0.80	1.05 ± 0.50	1.20 ± 0.20	0.98 ± 0.7
**5a**	50.4 ± 0.96	39.9 ± 0.52	19.2 ± 1.13	12.8 ± 0.62	>100	>100
**5b**	>100	>100	>100	>100	>100	>100
**5c**	54.9 ± 1.00	27.2 ± 0.75	15.70 ± 0.95	11.3 ± 0.63	>100	>100
**6a**	>100	>100	>100	>100	>100	>100
**6b**	>100	>100	>100	>100	>100	>100
**6c**	>100	>100	>100	>100	>100	>100
**7a**	>100	>100	>100	>100	>100	>100
**7b**	>100	>100	>100	>100	>100	>100
**7c**	>100	>100	>100	>100	>100	>100

### Human topoisomerases I and II inhibition assays

**Figure 2.**
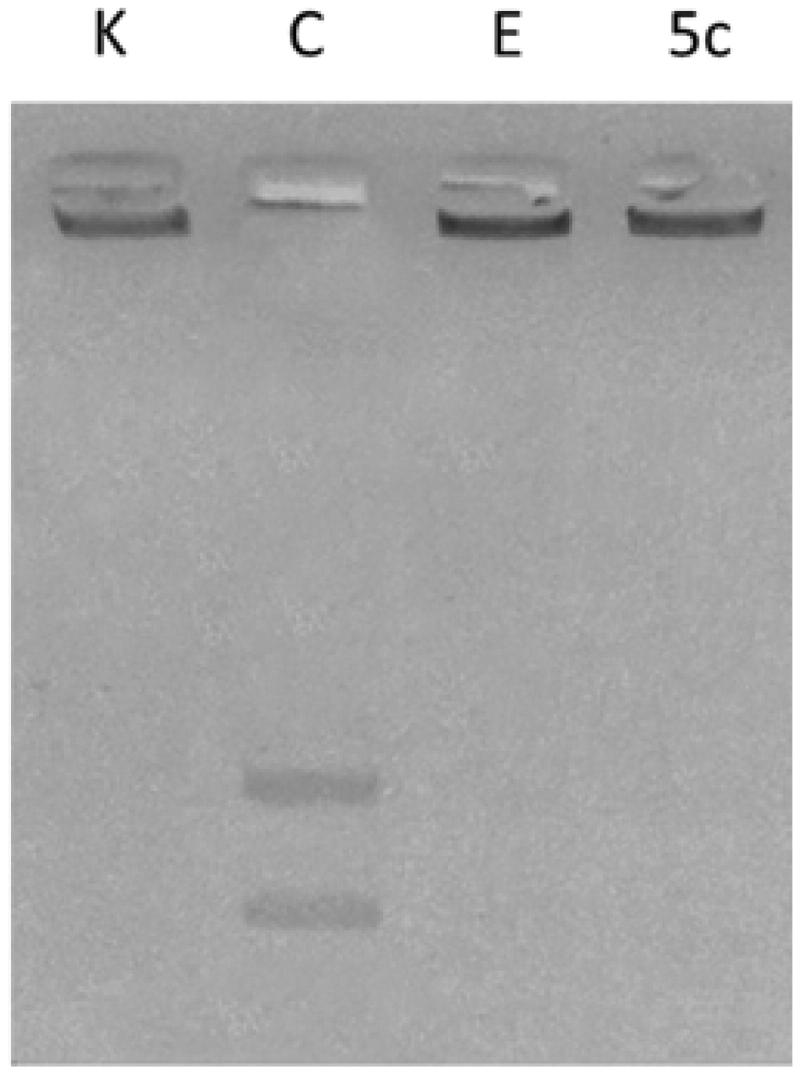
hTopoII inhibition assay. kDNA was incubated with human Topoisomerase II in the absence (lane C, vehicle DMSO) or presence of compound **5c** (lane 5c) or Ellipticine (lane E) at 10 and 50 µM, respectively. Lane K, kinetoplast DNA (kDNA).

Several studies reported that carbazole derivatives and Ellipticine are inhibitors of human Topoisomerases I and II. In particular, Ellipticine exhibits multi-modal mechanism of action with DNA intercalation and hTopoII inhibition^46–48^. For these reasons, the most active compound **5c**, structurally correlated to Ellipticine, was evaluated for its ability to inhibit DNA metabolising enzymes, i.e. hTopoI and II direct *in vitro* assays. The latter evidenced no inhibition of the hTopoI (data not shown) upon **5c** exposure, at least under the adopted experimental conditions. On the contrary, in hTopo II decatenation assays, the same compound was able to inhibit hTopoII activity at 10 µM concentration (Figure 2, lane **5c**), preventing the hTopoII from cutting the catenated kinetoplast DNA (KDNA), as instead happens in the experiment control (Figure 2, lane C) where the enzyme releases the nicked open circular minicircles and fully closed circular rings (decatenation products) visible at the bottom of gel as two distinct DNA bands. As well in these experiments, Ellipticine was used as reference molecule (Figure 2, lane E) at the concentration of 50 µM to obtain a complete inhibition^27^.

### Docking simulations

**Figure 3.**
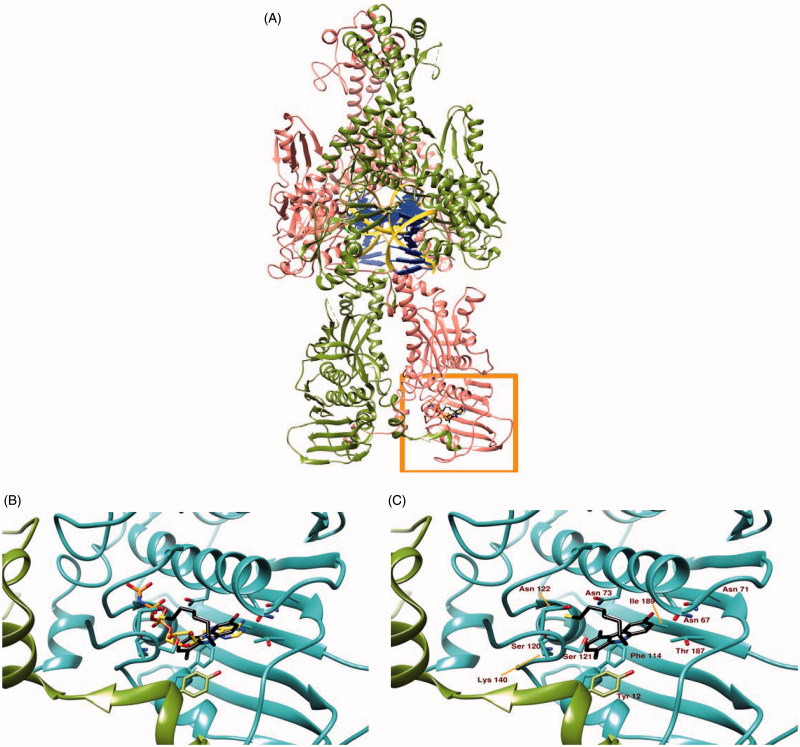
Panel (A) Ribbon representation of a dimeric hTopoII (chains A and B coloured in pink and olive green respectively) in complex with DNA (yellow ribbon and blue lego). The area within the orange rectangle highlights ATP binding site. Panel (B) A close up of the ATP binding site showing the different binding modes of AMP-PNP, a non-hydrolisable ATP analogue, (orange) and **5c** (black sticks) as suggested by docking simulations. Panel (C) shows the protein residues involved in **5c** binding.

The binding site of **5c** to hTopoII was identified by our docking simulation as coincident to the ATP-binding site (Figure 3, panel A), with the docked moiety almost perfectly superposed to the one of ANP-PNP (a non-hydrolysable ATP analogue), determined experimentally through X-ray diffraction (Figure 3, panel B). The binding site includes different amino acids from both chains of hTopoII, which contribute to binding stability mostly through polar and hydrophobic interactions (Figure 3, panel C). The best pose for molecule in the most populated cluster has a binding energy of −7.10 kcal/mol, as calculated by the server “Achilles”. A visual inspection of the crystallographic structure of hTopoII in complex with AMPPNP indicates that (1) the adenine moiety is hydrogen bonded to residues Asn 67, Asn 92 and Thr 187; (2) the ribose ring is stabilised by hydrogen bonds with Ser 120 and Ser 121; (3) the phosphates tail interacts with the peptidic nitrogen of Gly 138 and with the side chains of Asn 63 Ser 120, Asn 122, Lys 140, Gln 348 and Lys 350 through hydrogen bonds. Our simulation poses **5c** with the carbazole moiety almost superposed to the adenine of the crystallographic AMPPNP, with the bromine interacting with Asn 92 and Thr 187 through halogen bonds and the SH group forming a weak hydrogen bond to Asn 122. Residues Asp 66, Ile 97, Ile 113, Phe 114, and Ala 139 further contribute to stabilise **5c** binding mode to hTopoII by hydrophobic interactions.

### Compound 5c induces parp-1 cleavage

**Figure 4.**
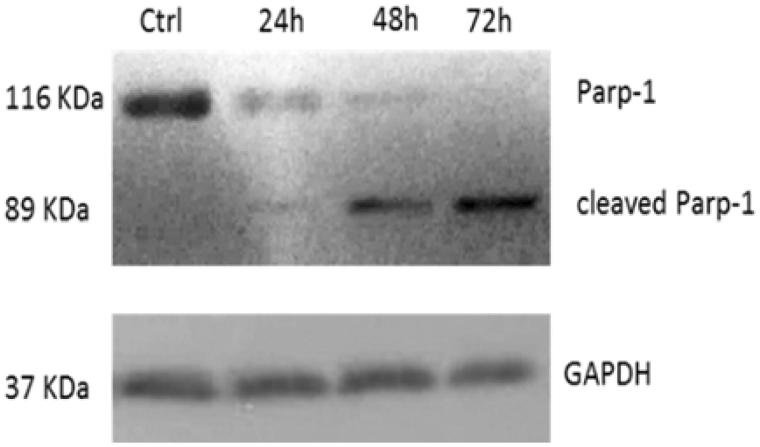
Parp-1 cleavage: time course of PARP-1 cleavage in uterine cancer Hela cells treated with **5c**, used at a concentration equal to its IC_50_ value. The control shown is referred to 72 h of vehicle exposure, but same results was obtained for 24 and 48 h. GAPDH was used as loading normalisation.

The observed inhibition of hTopoII pushed us to evaluate whether cells were undergoing cell death by apoptosis. The Topoisomerase II, indeed, plays an important role in DNA replication, transcription, recombination, thus its inhibition provokes genotoxic and mutagenic effects, leading to cell death. In response to DNA damage, cells may utilise the poly-(ADP) ribose polymerase (Parp-1), a nuclear enzyme that catalyses the transfer of the ADP-ribose portion of NAD^+^ into a specific subgroup of nuclear substrates^49^. Parp-1 is an important regulator of the DNA base excision repair (BER) pathway and it is involved in the maintenance of genomic integrity and survival following genotoxic insults. Parp-1 is specifically proteolysed by executioner caspases (caspases 3 and 7) to an 89 kDa catalytic fragment during the final steps of apoptosis^50^ ^,^ ^51^. Indeed, Parp-1 cleavage promotes apoptosis by preventing DNA repair-induced survival and across the block of energy depletion induced by necrosis^52^. With this in mind, we treated HeLa cells with compound **5c** at a concentration of 10 µM and at three different times (24, 48 and 72 h). At the end of the treatment, the cells were lysed and the total cellular protein content was extracted and used for western blot analyses. The obtained results, reported in Figure 2, showed the presence of the cleaved form of Parp-1 (about 89 KDa) in cells treated with compound **5c**, visible at 24 h and increased at 48 and 72 h, together with a decrease of the native protein, due to the cleavage. At 72 h, the only appreciable form is the cleaved one, whereas, on the contrary, in vehicle-treated cells only the whole form is present and not the cleaved one. In Figure 4, the shown control is referred only to 72 h, but same results were obtained at 24 and 48 h (data not shown).

**Figure 5.**
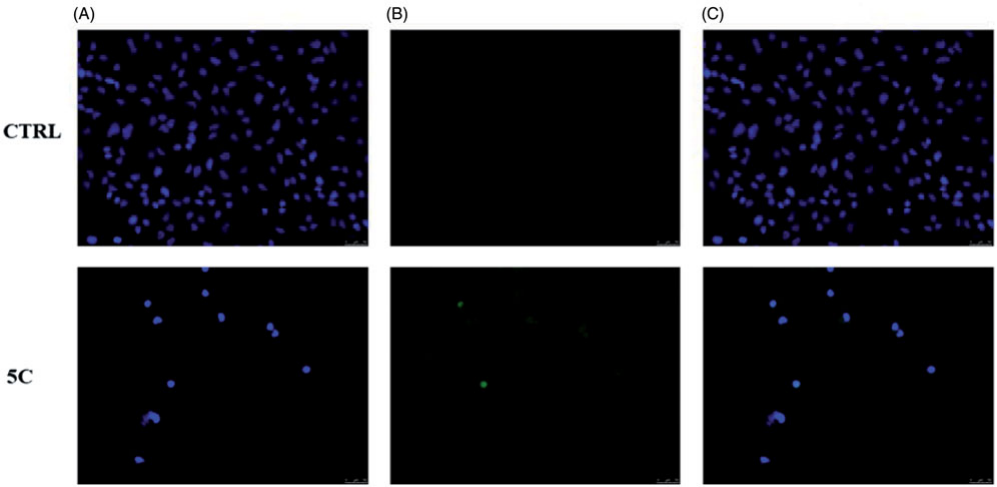
TUNEL assay of HeLa cells. Cells were treated for 24 h with compound **5c** (at the concentration equal to its IC_50_) or with vehicle (CTRL). Then they were fixed, subjected to TdT reaction, washed, dyed with DAPI and imaged under a fluorescence microscope (20× magnification, excitation/emission wavelength 490 nm/515 nm for CF™488 A, panels B, and 350 nm/460 nm for DAPI, panels A). Panels C show the merge.

In order to assess whether the observed cytotoxic effects could depend on the induction of apoptosis under treatment, we exposed Hela cells to **5c** compound, at the concentration equal to its IC_50_ value for 24 h. As in Figure 5, a green nuclear fluorescence (**5c**, panel B) indicates DNA damage and that cells have undergone the apoptotic process due to the exposure to the **5c** compound, whereas no fluorescence resulted in the vehicle-treated cells used as experiment control (Figure 5, CTRL, panel B). These data confirm that the selected compound induced DNA damage and led to cell death triggering the apoptotic mechanism.

### Compound 5c induces apoptosis by the intrinsic pathway

**Figure 6.**
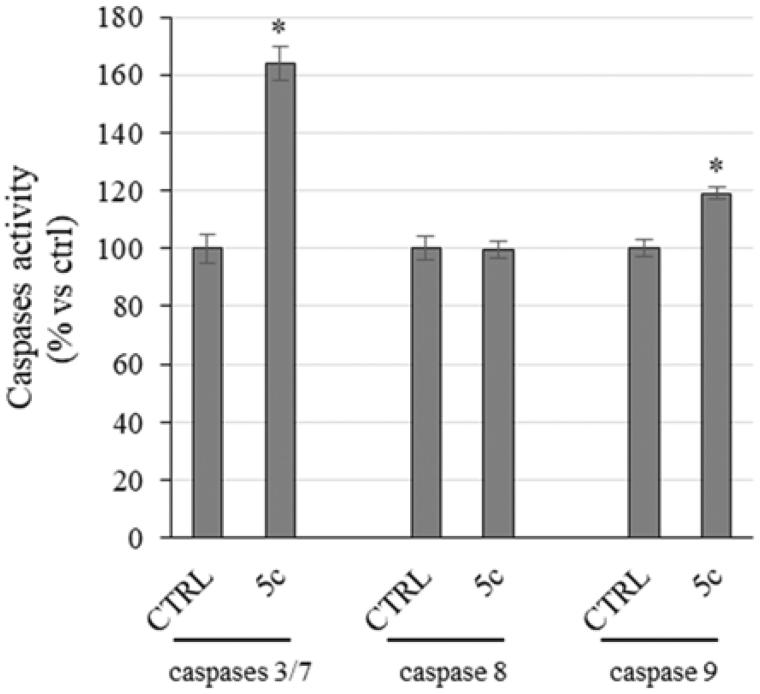
Caspases activity. Activation of caspases 3/7 and 9 following to the treatment of HeLa cells with the compound **5c** at a concentration equal to its IC_50_ value for 24 h. Columns mean, bars SD, *p < .001.

Even though the mechanisms of apoptosis are complexes and involve an energy-dependent cascade of molecular events, it is noteworthy that there are at least two main pathways,^53^ not necessarily independent from each other, i.e. the extrinsic or death receptor pathway and the intrinsic or mitochondrial one^54^. In order to establish which pathway was involved, we evaluate the activity of initiators caspases, namely caspases 8 and 9, that are involved, respectively, in the extrinsic and intrinsic pathway, and effectors or executioner caspases 3 and 7, by means of a luminescent assay. Caspases are usually expressed in an inactive form and after activation they may initiate a proteolytic cascade,^55^ in which one caspase may activate other caspases or cleave cell proteins, included Parp-1, amplifying the apoptotic cell death^53^. Effectively, we noticed a little, but significant, rise of caspase-9 activity in HeLa cells treated with compound **5c** for 24 h, whereas no increase of caspase-8 activity was evidenced (Figure 6). Moreover, we observed a clear increase of caspases 3 and 7 activity (Figure 6), which are cleaved by the initiator caspase-9 and that, in turn, may activate other pro-apoptotic proteins and cleave Parp-1, as we already observed (Figure 4). Summing up, the exposure of HeLa cells to compound **5c** induces the activation of caspase-9 that activates caspases 3/7, responsible of the observed Parp-1 cleavage that provokes the inability of DNA reparation processes and leads to cell death by apoptosis.

**Figure 7.**
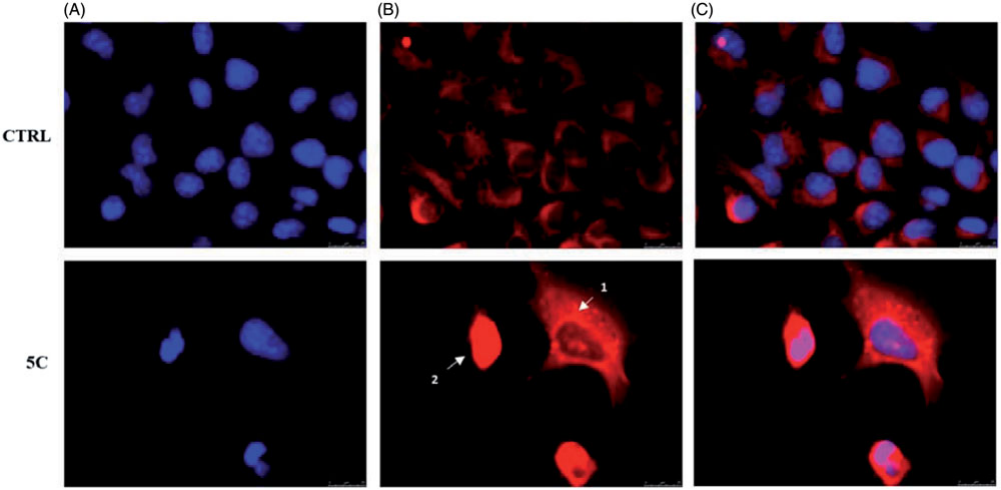
Cytochrome c release during apoptosis in Hela cells. In vehicle-treated cells Cytochrome c is localised within the mitochondria (panel B, CTRL). During the early stages of apoptosis, induced by compound **5c** treatment at the concentration equal to its IC_50_ for 24 h, mitochondria fragmentation occurs (as shown by the red dots indicated by arrow 1 in panel B, **5c**) and Cytochrome c is partially released. In the last apoptotic cell stage, a full release of Cytochrome c is observed (diffused red fluorescence, indicated by the white arrow 2). Panel A, DAPI (excitation/emission wavelength 350 nm/460 nm), panel B, Alexa Fluor® 568 (excitation/emission wavelength 578 nm/603 nm), panel C, overlay. Images were acquired at 63× magnification and are representative of three separated experiments.

Another important event of the intrinsic apoptotic pathway is represented by the release of Cytochrome c from mitochondria to the cytosol^56^. Thus, we wanted to evaluate the localisation of Cytochrome c, by means of immunofluorescence assays in HeLa cells treated with the compound **5c** for 24 h at the concentration equal to its IC50, and fixed (see experimental section for details). These assays confirmed that compound **5c** determines the release of Cytochrome c from mitochondria to the cytosol (Figure 5). Indeed, the fluorescence (red colour in Figure 5) is diffused and delocalised throughout the cell cytoplasm, whereas in vehicle-treated cells (CTRL), the fluorescence is strictly localised in well-defined areas of the cell, given that Cytochrome c is normally localised in the mitochondrial compartment. In Figure 5, it should be evidenced how it is possible, as already reported^57^, to identify the different stages of mitochondria disruption during apoptosis. In fact, a punctiform fluorescence in the cytosol (white arrow 1) indicates mitochondria fragmentation and partial release of Cytochrome c into the cytosol, which occurs during the early stages of apoptosis. Successively, the release of Cytochrome c is complete (white arrow 2, Figure 7) and the fluorescence (red) is greatly increased and spread throughout the cytosol. On the contrary, in cells treated with the vehicle only, the fluorescence (red) is defined and organised in an ordered and filamentous way, indicating that mitochondria are intact.

## Conclusions

Cancer is one of the leading causes of death in economically developed countries, probably due to population aging and lifestyle choices, such as smoking, lack of physical activity and unbalanced diets^58^. Among the human cancers, breast and uterine cancers represent the death cause of almost the 26% and 7% of women, respectively^59^. Even though the earlier diagnosis, as result of the publicity surrounding the disease, and its prevention increased the survival, the availability of new and improved chemotherapies facing the resistance occurrence and the toxic side effects is still a major challenge. Under this point of view, the preclinical research may offer a panel of new potential anticancer molecules that may be useful as valid alternative to the classic therapies or in co-treatment. Herein, we have studied a series of new *N*-thioalkylcarbazole derivatives, structurally correlated to the most known Ellipticine, which exert interesting antiproliferative effects on two *in vitro* models of the most common cancer types in women. Our outcomes indicates that two derivatives, **5a** and **5c**, possess a good antitumour activity against two models of breast cancer cells and a higher selectivity toward the cervical and endometrial cancer cells. Even though Ellipticine, the ancestor of carbazoles, possess a higher, but non-selective, activity compared with these derivatives, it should be evidenced how these properties come together with very high cytotoxic effects on non-tumoural cells used in these studies. On the contrary, the two lead molecules, did not affect the viability of non-tumoural cells, at least at the doses lower than 100 µM. The underlying molecular mechanism by which the most active compound exerts its antiproliferative activity is represented by the inhibition of hTopoII and the activation of the mitochondrial or intrinsic apoptotic pathway. Indeed, the treatment of HeLa cells with compound **5c** induces caspase-9 activation, which targets several proteins, amongst them caspases 3 and 7, known as executioner caspases. The latter are able to continue the cascade of proteolytic cleavages, as we observed for Parp-1, one of the most important protein involved in DNA damage repair. Since Parp-1 is cleaved, cells are unable to repair their DNA, which increased damage, together with hTopoII inhibition, is the major stimuli that direct cells to die by apoptosis. Effectively, Hela cells treated with **5c** are clearly undergoing apoptosis, as demonstrated by TUNEL assay and, particularly, through the mitochondrial mechanism. Indeed, immunofluorescences studies evidenced as Cytochrome c is released from its natural site, namely mitochondria, to the cytosol of treated Hela cells. Considered the biological features of compound **5c**, we are confident that this lead compound may be a useful and interesting tool in the treatment of uterine cancer.
